# *Operando* NRVS on LiFePO_4_ Battery with ^57^Fe Phonon DOS

**DOI:** 10.3390/cryst15100841

**Published:** 2025-09-27

**Authors:** Alexey Rulev, Nobumoto Nagasawa, Haobo Li, Hongxin Wang, Stephen P. Cramer, Qianli Chen, Yoshitaka Yoda, Artur Braun

**Affiliations:** 1 Laboratory for High Performance Ceramics, Empa. Swiss Federal Laboratories for Materials Science and Technology, Überlandstrasse 129, CH-8600 Dübendorf, Switzerland; 2 SETI Institute, Mountain View, CA 94043, USA; 3 Global College, Shanghai Jiao Tong University, Shanghai 200240, China; 4 Precision Spectroscopy Division, SPring-8/JASRI, Sayo, Hyogo 679-5198, Japan

**Keywords:** NRVS, vibrational spectroscopy, phonon DOS, LiFePO_4_, lithium ion battery, deep learning

## Abstract

The vibration properties of materials play a role in their conduction of electric charges. are Ionic conductors such as electrodes and solid electrolytes are also relevant in this respect. The vibration properties are typically assessed with infrared and Raman spectroscopy, and inelastic neutron scattering, which all allow for the derivation of the phonon density of states (PDOS) in part of a full portion of the Brioullin zone. Nuclear resonant vibration spectroscopy (NRVS) is a novel method that produces the element-specific PDOS from Mössbauer-active isotopes in a compound. We employed NRVS *operando* on a pouch cell battery containing a Li^57^FePO_4_ electrode, and thus could derive the PDOS of the ^57^Fe in the electrode during charging and discharging. The spectra reveal reversible vibrational changes associated with the two-phase conversion between LiFePO_4_ and FePO_4_, as well as signatures of metastable intermediate states. We demonstrate how the NRVS data can be used to tune the atomistic simulations to accurately reconstruct the full vibration structures of the battery materials in *operando* conditions. Unlike optical techniques, NRVS provides bulk-sensitive, element-specific access to the full phonon spectrum under realistic *operando* conditions. These results establish NRVS as a powerful method to probe lattice dynamics in working batteries and to advance the understanding of ion transport and phase transformation mechanisms in electrode materials.

## 1. Introduction

Lithium iron phosphate (LFP) with an olivine structure is one of the main cathode materials for lithium-ion batteries. The material has a theoretical capacity of 170 mAh/g, and a capacity of 120 mAh/g can be achieved since over 25 years ago [1]. One of the main advantages that make the olivine cathode an attractive material is the improved stability compared to the layered cathodes like nickel–manganese–cobalt oxides [2]. One of the drawbacks of olivine cathodes is the slower cycling rate, which is limited mostly by solid-state diffusion within the olivine structure. Lithium diffuses through 1D channels framework in olivine structure, which is generally considered slower than 2D diffusion in layered oxides.

The ionic diffusion in solids, especially in case of light ions, is a complex process involving many parameters. For example, it was proposed that some vibration modes of the lattice may propel the ionic transport by dynamically lowering the jump barrier. It was shown that low-energy phonons in an olivine structure enhance lithium diffusion, allowing the higher cycling rate [3]. Generally, the lower phonon frequencies suggest softer lattice and enhanced ionic transport, so phonon density of states can be used as a predictor for ionic conductivity [4,5]. Therefore, understanding the vibration properties of the cathode material is crucial for understanding the solid-state mass transfer phenomena and for designing the materials for fast charging devices.

Study of vibration properties of battery materials, especially in *operando* conditions, is a complex task. The most convenient techniques, like optical (Raman or infrared, IR) vibrational spectroscopy, lack penetration depth, so they require notable modifications of the cell and material to make acquisition of the signal possible. Such modifications, like holes in the current collector or separator, often lead to non-uniform current distribution and distorted signal [6]. Moreover, the carbon coating, which is common in LFP cathodes, was shown to block the Raman and IR signal from iron phosphate [7]. In addition, the information obtained from IR or Raman spectra gives only limited insights into the phonon structure, as it only shows some modes around the gamma-point of the Brillouin zone. Techniques like inelastic neutron scattering can be used to study the vibrational properties of active materials. It was demonstrated that the difference between surface and bulk phonons, measured with inelastic neutron scattering (INS), allows us to obtain some insights on morphology of the active particles [8]. Despite the great penetration depth of neutrons, the technique is not very well suited for *operando* conditions due to the multiple components of the battery that obfuscate already complex signals.

Thanks to the presence of iron in the structure, LFP opens the possibility to use techniques based on nuclear resonance, like Mössbauer spectroscopy. Such techniques suit perfectly for *operando* experiments due to a combination of signal penetration depth and element specificity, which guarantees that the signal comes exclusively from the cathode material. The nuclear resonance vibrational spectroscopy (NRVS) is a synchrotron-based technique that allows to study the vibrational density of states, projected on a Mössbauer-active element [9]. This technique was used to study the dynamics of active centers in biological systems [10], or the phonon structure of crystalline solids [11]. Here, we for the first time demonstrate the *operando* study of the phonon structure of an active cathode material using the NRVS technique during the cell cycle.

## 2. Materials and Methods

### 2.1. Synthesis

**Figure 1.**
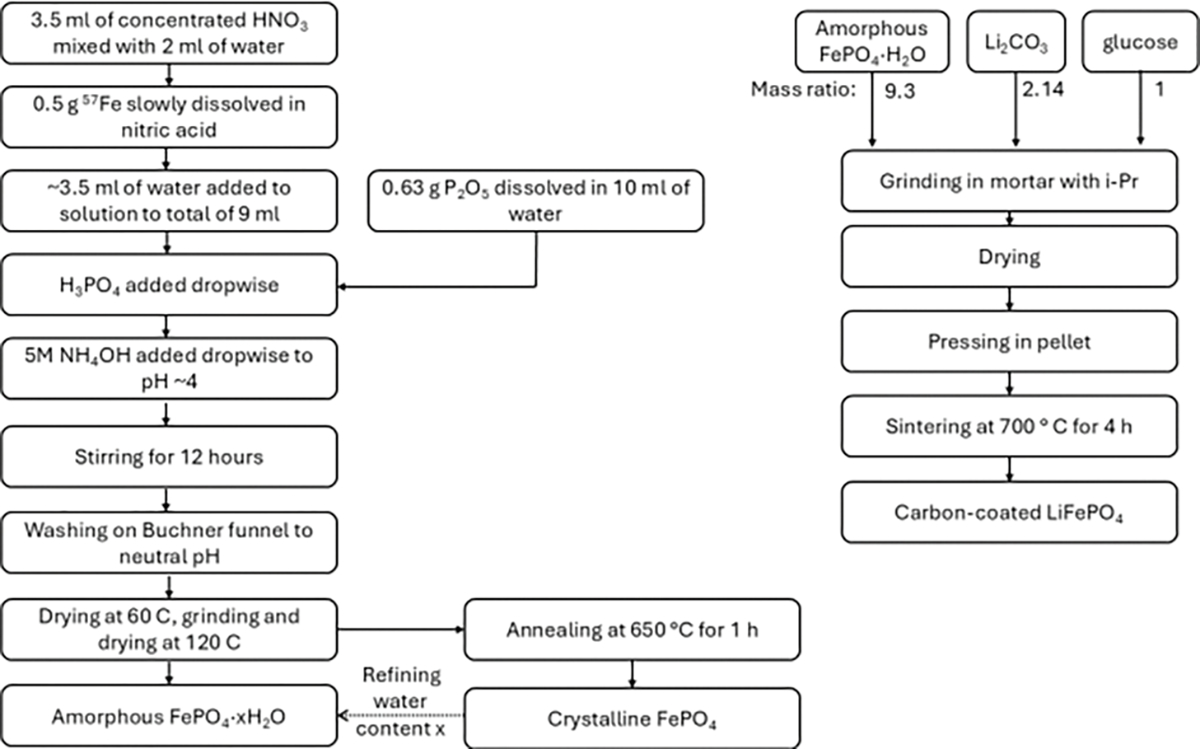
Flow diagram for our synthesis of iron phosphate and lithium iron phosphate with ^57^Fe isotope metal sheet as raw material.

^57^Fe-enriched LFP was synthesized using the method modified from [12]. We need to mention here that the ^57^Fe isotope is rarely available as a salt or oxide. We were therefore required to develop our own synthesis route, illustrated in the flow diagram in Figure 1, based on available iron metal. As a source of the ^57^Fe isotope, ^57^Fe-enriched metal chips (Neonest AB (BuyIsotope.com), SE-171 50 Solna, Sweden) were used, the isotope contents of which are detailed in Table 1, and according to which the ^57^Fe content is larger than 96%.

A total of 3.5 mL of concentrated HNO_3_ were mixed with 2 mL of deionized water, and 0.5 g of ^57^Fe were dissolved in the nitric acid. After the complete dissolution, water was added to a total of ~9 mL of solution. Separately, 0.63 g of P_2_O_5_ (Sigma-Aldrich) were slowly dissolved in 10 mL of water to obtain the H_3_PO_4_ solution. Then, the H_3_PO4 solution was added dropwise to the iron nitride solution under intensive stirring. Then 5M NH_4_OH solution was slowly added to the mixture under stirring and heating at 50 °C until the pH reached a value of ~4, when a thick gel-like sediment was formed. The mixture was stirred for at least 12 h, then filtered on Buchner vacuum funnel and washed with deionized water until neutral pH. Then, the sediment was dried at 60 °C, ground in a mortar, and dried again at 120 °C for 5 h. This resulted in an amorphous FePO_4_·xH_2_O that does not show any Bragg reflections. A portion of amorphous FePO_4_·xH_2_O was annealed at 650 °C for 1 h to estimate the amount of water and measure Raman spectra of delithiated FP. After annealing the amorphous FP crystallizes and the sample demonstrates narrow XRD peaks, corresponding to the FePO_4_ crystalline rodolicoite phase. The mass loss after annealing was 11%, so the estimated amount of water in amorphous powder was x = 1.

Stoichiometric amounts of amorphous FePO_4_·H_2_O and Li_2_CO_3_ (5% excess) with 8 wt.% of the total mixture mass of glucose (final mass ratio: FePO_4_·H_2_O/Li_2_CO_3_/glucose = 9.3: 2.14: 1) were mixed in a mortar with isopropanol and thoroughly ground, then dried at 60 °C for 1 h. Then the mixture was pressed in a pellet and sintered at 700 °C for 4 h in nitrogen atmosphere resulting in black pellet. The sintered pellet was then crushed and ground in a mortar, resulting in LiFePO_4_ particles covered with carbon. The phase composition was validated with XRD, demonstrating the LFP phase with a small amount of impurities.

### 2.2. Pouch Cell Battery Assembly

The obtained powder was assembled to a battery positive electrode as follows. To prepare the slurries, LFP powder was mixed with polyvinylidene fluoride dissolved in N-methyl-2-pyrrolidon (NMP) and carbon black (super C-65) in a mass ratio 90:5:5 and mixed in a planetary mixer. The slurries were cast on Al foil with a doctor blade applicator, dried at 80 °C in air, then pressed and vacuum-dried in the antechamber of the glovebox. The resulting active material mass loading was approximately 8 mg/cm^2^.

**Figure 2.**
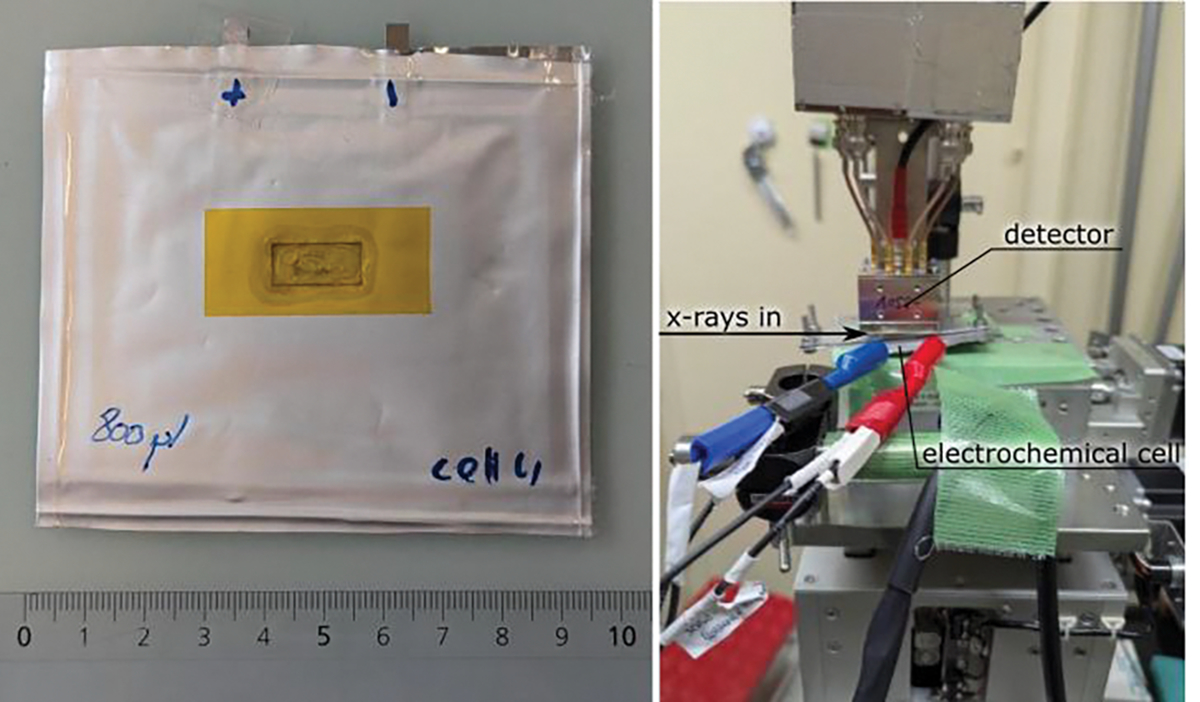
(**Left**) photo of the *operando* cell with Kapton window. (**Right**) photo of the cell in the endstation of the beamline BL19LXU at SPring-8.

The cells were assembled in a glove box in an argon (Ar) atmosphere. As a counter electrode, graphite on Cu foil was used (active material loading ~12 mg/cm^2^). The electrodes were separated with a Whatman glass-fiber separator, and approximately 800 μL of 1M LiPF_6_ solution in a mixture of ethylene carbonate/diethyl carbonate/dimethyl carbonate (1:1:1 mass) were added as the electrolyte. Cells were assembled in a pouch case with a 7 × 14 mm window covered with Kapton tape. During the experiment, the cell was clamped with an aluminum frame to ensure tight contact between the electrodes. The photo of the cell and the placement of the cell in the beamline are displayed in Figure 2.

### 2.3. Battery Charging and Discharging

The cell was cycled with IVIUM Vertex.100mA potentiostat at a constant current at a rate of approx. C/30 (50 μA/cm^2^). After reaching the threshold potential of 4.2 V, the cell was held potentiostatically for ~1 h at this potential and then discharged at the same rate. This allows us to determine the capacity of the pouch cell as a diagnostic quantity by integrating the current (compare ref. [13]) with respect to the cycling curve, as the total amount of charge during the charging cycle. The specific charging protocol was the following: charging at a constant current until the threshold voltage is reached, then further charging at a constant voltage (equal to the threshold value) until the current drops to lower than 10% of the initial current. Then, the value of the total charge to this point was divided by the active material mass. The obtained values are 136.7 mAh/g on charge, and 129.3 mAh/g on discharge (~5% capacity loss). Since this is the first cycle of the cell (formation cycle), when the solid electrolyte interfaces are formed, some irreversible capacity loss is anticipated. Therefore, we consider a nominal capacity to be around 135 mAh/g.

### 2.4. Nuclear Resonance Vibrational Spectroscopy

Nuclear resonance vibrational spectroscopy was performed at beamline BL19LXU at Spring-8 in Hyogo, Japan [14,15]. The incident X-ray energy was set around 14.413 keV to excite the γ-transition of ^57^Fe [16]. The spectra were acquired in the range from approximately −42 to +65 meV from the elastic peak. Spectra were acquired continuously in *operando* mode, with each spectrum acquisition taking approximately 20 min. This means that the state of charge of the battery was not kept constant, while the NRVS scans were in course. NRVS spectra were processed using “NRVS tool” from spectra.tools [17] and PHOENIX software [18] to obtain the Fe-projected phonon density of states (PDOS).

We briefly explain how an NRVS spectrum is generated [9,17]. The battery cell containing ^57^Fe is scanned (this is not a scan over position, but a scan of the energy) with the X-ray beam energy in the aforementioned range covering the nuclear γ-transition at E_1_ = 14.413 keV of ^57^Fe, with vibrational levels included. This results in nuclear back radiation of scattered energy E_2_ = hν_1_, comparable to the scheme on the left of Figure 1 in our previous publication [11]. There is also X-ray fluorescence from the K shell electrons of ^57^Fe by internal conversion with energy hν_2_. The X-ray intensities at hv1 and hv2 are recorded versus the vibration energy E_vib_ = E_1_−E_2_ = 14.413 keV−hν_1_. The resulting spectrum is comparable with an optical Raman spectrum with elastic peak and Stokes and anti-Stokes shifted peaks, but it only probes the ^57^Fe.

### 2.5. Optical Raman Spectroscopy

Optical Raman spectra of the carbon-coated LFP powder, the LFP powder cast on Al foil and annealed FP powder were obtained using a Renishaw inVia Qontor spectrometer. The laser wavelength was 532 nm, with 10 s exposure time, 6% power (1.9 mW), and 5 accumulations. A representative spectrum is shown in Figure 8. For comparison, the Raman shifts in LFP and FP agree well with the characteristic peaks documented and explained in the publications by Wu et al. [19] and Zhang et al. [20], respectively.

### 2.6. Calculation of Total and Partial Vibrational Density of States (PVDOS)

Phonon properties were simulated using a deep-learning atomistic model MatterSim [21] with the pre-trained model MatterSim-v1.0.0–5M, available in MatterSim repository. As the starting configuration for LFP calculations, the LFP structure from the Materials Project mp-19017 was used [https://doi.org/10.17188/1193803]; for FP calculations, lithium atoms from the same structure were removed. To obtain the Fe-projected phonon density of states (PDOS), the structures were relaxed until all the forces acting on the atoms were less than 10^−5^ eV/Å, and the phonon structure was calculated using finite displacement method using PHONOPY code [22,23] with non-analytical term correction (NAC) applied. For phonon calculations, 3 × 3 × 3 supercells were constructed, and the displacement with the amplitude of 0.01 Å was applied. To calculate the phonon DOS, the 15 × 15 × 15 mesh of k-points was used for sampling the Brillouin zone. The dielectric constant and Born effective charges for the NAC were calculated using Quantum ESPRESSO package [24–26]. Compared with the experimental phonon DOS, the Gaussian broadening was applied to the calculated phonon DOS with the width derived from the elastic peak in the experimental spectra, which was taken as a resolution function.

## 3. Results and Discussion

### Crystallographic Structure

**Figure 3.**
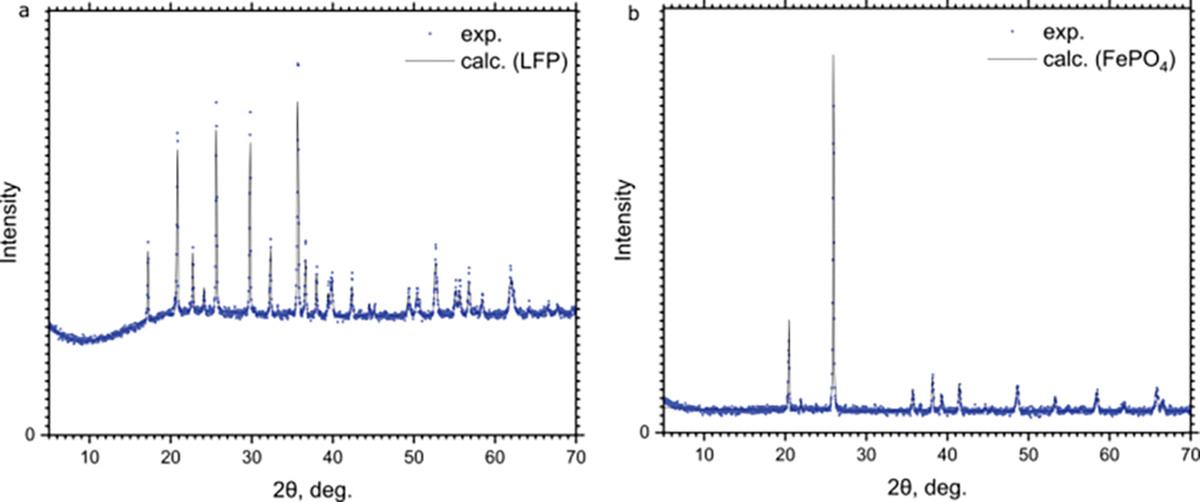
(**a**) X-ray diffractograms of SFP and (**b**) FP. The measured data points and the profile from Rietveld refinement are shown.

The X-ray diffractograms of the pristine LFP material show in Figure 3 that the samples are phase-pure LFP. The diffraction patterns of both LFP and intermediate FePO_4_ in rodolicoite phase match those published in [12]. It should be noted that the FePO_4_ obtained by traditional ceramic synthesis, such as sintering the amorphous FePO_4_·xH_2_O precursor, crystallizes in a different phase than FP synthesized by electrochemical delithiation.

Rietveld refinement shows a slight ~10% deficiency in lithium positions, which may be associated with lithium sublimation during the annealing process [27], despite the excess of lithium carbonate. It is therefore plausible that the cells demonstrate a slightly lower total capacity (~135 mAh/g) than the usually observed values and matches those reported for LFP, synthesized by the same coprecipitation method [12]. The occupancy of all sites was determined by Rietveld refinement and is listed in Table 2.

**Figure 4.**
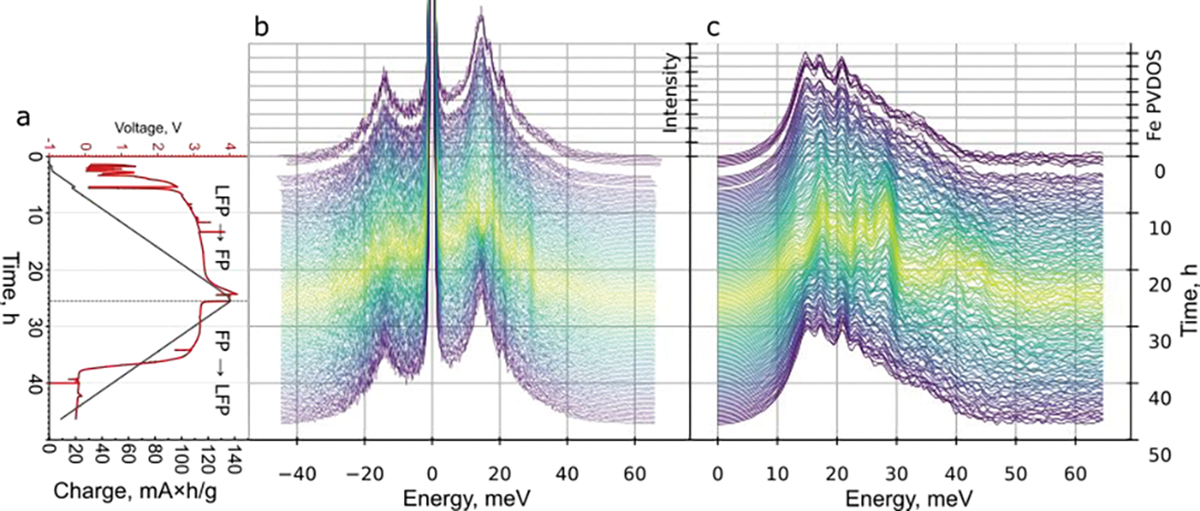
(**a**) Voltage profile of the cell during *operando* experiment and the total charge passed through the cell. (**b**) Raw NRVS spectra, collected from the cell. The spectra were centered so that the energy of the elastic peak is zero. (**c**) Fe-projected partial vibrational density of states, derived from the NRVS spectra. Color of the spectra corresponds to the state of charge.

Figure 4a shows the electrochemical cycling curve (voltage profile) of the cell, the corresponding NRVS *operando* spectra, and the derived Fe-projected partial vibrational DOS (PVDOS). Inscribed in the curve is the qualitative range where the electrode constitutes LFP and FP. At the beginning of the charging, the electrode is fully LFP. At the fully charged state, we expect the material to be fully delithiated and thus FP. The corresponding change in the oxidation state of Fe is, for example, illustrated in Figure 5 in ref. [28]. Our cycling curve shows a slight slope at the beginning of charging and an accelerated voltage drop in discharge before the end of charge at the ~35 h mark. We speculate that this observation might come from moisture from the outside, which may have leaked into the cell through the Kapton window, which is known to be permeable for water [29–32]. Nevertheless, the NRVS spectra show the reversible behavior with the changes still happening after the voltage drop at ~35 h. This indicates that the lithiation of LFP continues, which can be explained by extraction of lithium from the electrolyte due to water-related loss in the lithium inventory.

The NRVS spectra show reversible changes upon lithiation and delithiation, which correspond to the two-phase conversion:


$${\text{LiFePO}}_{4}\to {\text{Li}}^{+}+{\text{FePO}}_{4}+{\text{e}}^{-}$$

**Figure 5.**
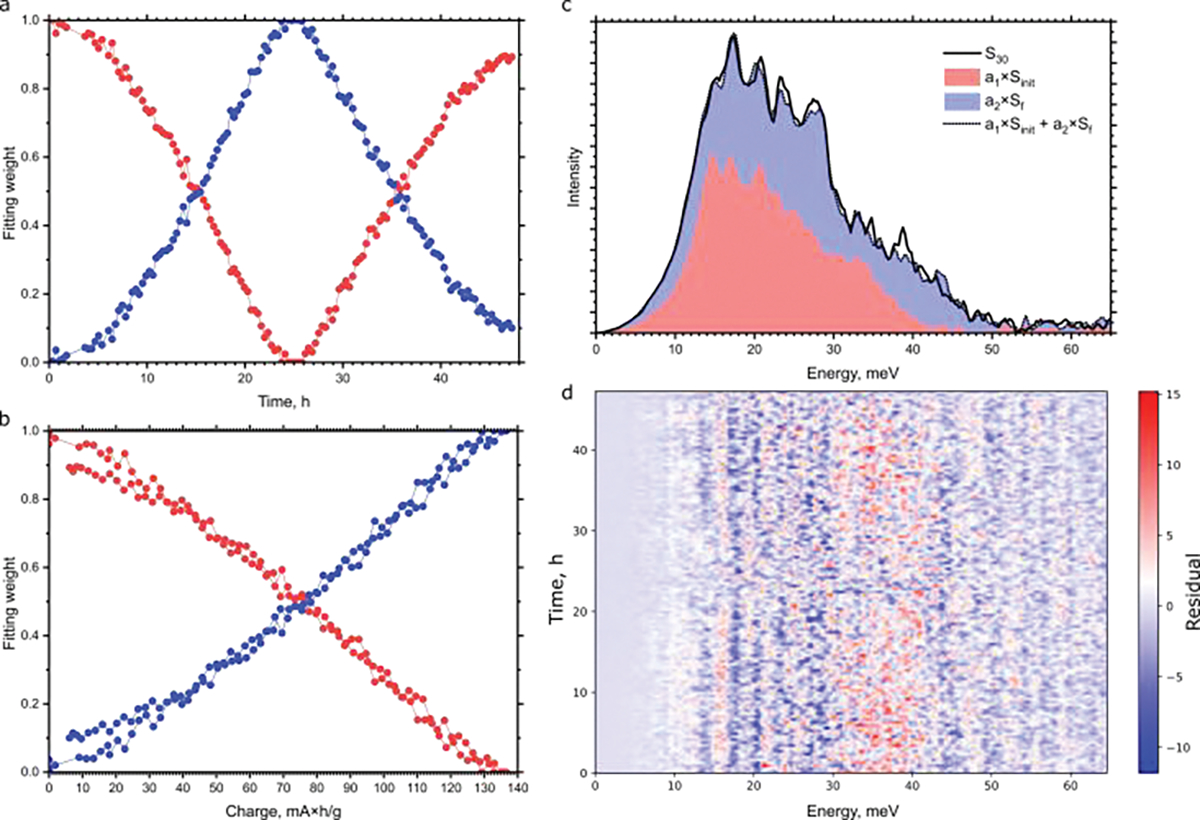
Weights a1 and a2 of fitting all experimental DOS versus time (**a**) and charge (**b**). (**c**) Example of fitting an intermediate PVDOS (S_30_) with a linear combination of initial and final PVDOS. (**d**) Residuals of the fitting of all spectra as a function of time of measurement from the start of experiment.

Figure 4c displays the Fe-projected PVDOS, obtained from the NRVS spectra. The phonon DOS clearly demonstrates the decrease in the peaks at ~15 and 20 meV and appearance of peaks at ~29 and 35–45 meV upon delithiation. We used the PVDOS in the initial state and the PVDOS obtained at the end of charging as the basis components and fitted all the intermediate PVDOS with a linear combination of the two basis components. The coefficients as a function of time and charge are shown in Figure 5. The linear relation between charge and the fraction of components indicates that all the intermediate spectra are at large composed of two phases that represent fully lithiated and delithiated states, which are ${\text{LiFePO}}_{4}$ and ${\text{FePO}}_{4}$. Figure 5d shows the residuals of the least-squares fit, obtained by the following equation:

$$R_{i}=\;S_{i}-\left(a_{1}\;\cdot \;S_{init}+\;a_{2}\;\cdot \;S_{f}\right)$$

where $S_{i}$, $S_{init}$ and $S_{f}$ are i-th PVDOS, initial PVDOS in fully lithiated state (LFP), and the final PVDOS in the fully charged (delithiated, FP) state, respectively, $a_{1}$ and $a_{2}$ are the coefficients shown in Figure 5a,b. The residuals show a systematic pattern for intermediate states (Figure 5d). The pattern looks symmetrical for the charge and discharge half-cycles, what indicates that it does not originate from the change in the state during the spectrum acquisition but is an indicator of presence of more phases [21] than just FP and LFP. It was previously reported that the conversion from LFP to FP goes via the metastable intermediate phase [33,34], which was detected with NRVS. At a certain state of charge, the notable change in the pattern can be observed: for example, the peak at ~16 meV disappears, and the peak at 20 meV appears. Based on the observed reversible change in the pattern, we cannot rule out more than one intermediate phase. Confirmation of such scenario would require further study.

**Figure 6.**
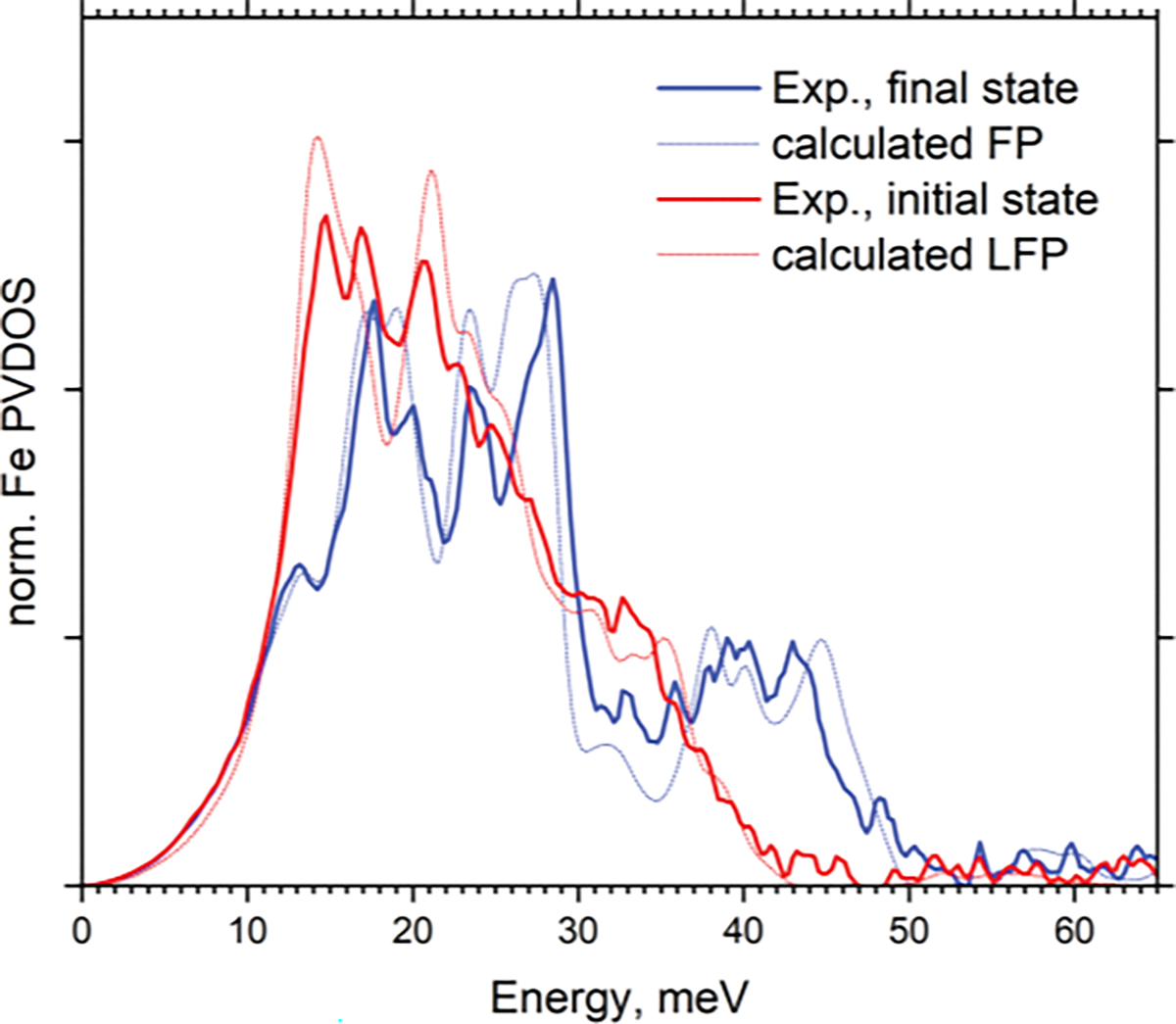
Experimental (solid lines) and calculated (dotted lines) PVDOS of Fe in LFP and FP.

Figure 6 displays the comparison between the experimental Fe PVDOS in fully charged and fully discharged states and calculated PVDOS of FP and LFP. While for the FP phase, the perfect agreement between the experimental and calculated data was achieved immediately, for the LFP, the best fit was achieved by stretching the energy scale of calculated data by a factor of 1.064. Application of such scaling is usual for the simulated vibrational spectra [35] and is justified by the semi-empirical nature of DFT (the choice of exchange-correlation functional and Hubbard U parameters). The interatomic potential MatterSim [21], that was used in our calculations, was trained on the DFT calculations and generally reproduces the results of DFT calculations with a high precision [36], so it inherits all the same issues and the same approach is justified in our case. This way, NRVS here was used to tune the model to the experimental data. Such tuning is additionally verified by the good fitting of the experimental Raman spectrum, as shown in Figure 5. For example, the highest peak at ~118 meV, which corresponds to the vibrations of PO_4_ tetrahedra and is not directly related to Fe atoms, is also precisely reproduced by the simulation, fitted to experimental NRVS. Our experimental PVDOS lends empirical confirmation to our calculations and support also those DFT calculations, which are reported in the literature [8].

The calculated PVDOS of FP reproduces the experimental one with great accuracy, reproducing all the observed features. The PVDOS of LFP, however, shows slight deviation between the experiment and calculations, with a peak at ~17 meV not reproduced by calculations. This may be attributed to the presence of Li vacancies in the initial state, which is not fully lithiated, which may introduce additional features to the phonon structure. The same phenomenon led to the systematic error, shown in Figure 3d, which is presumably produced by the presence of the third intermediate phase, which may also have some ordered Li vacancies, and whose phonon spectrum cannot be described as a linear combination of the fully lithiated and fully delithiated phases.

Figure 5 shows the experimental optical Raman spectrum of LFP and the NRVS-derived PVDOS in comparison with the calculated phonon structure. The Raman spectrum agrees with the reported in the literature in situ [19,37] or ex situ [20] spectra. For the orientation of the reader: the prominent peak at below 120 meV corresponds to the symmetric stretch vibration of the phosphate cage PO_4_^3−^ with ν_1_ ≈ 950 eV. Due to the experimental constrains, the in situ Raman spectra reported in the literature only allows the highest peak at ~950 cm^−1^ (=118 meV) that corresponds to the relatively flat band to be observed, associated with PO_4_ tetrahedra vibrations with A_g_ symmetry. The two smaller peaks at 123 meV and 132 meV are the asymmetric stretch of the same structure. The vibrations of PO_4_ octahedra obviously are sensitive to the presence of lithium in the crystal structure, allowing the lithium content in the material to be monitored.

The vibrations associated with Fe all have lower energy and are confined below ~50 meV (≈400 cm^−1^, this is around the two well developed peaks at 22 and 35 meV, corresponding to 220 and 290 cm^−1^ with translation or Fe and coupled translation and vibration of the phosphate cage), so the *operando* NRVS focuses on the different regions of the vibration structure. Wu et al. find this region interesting because of the prominent differences between lithiated and non-lithiated material [19]. It was shown that vibrations in this region may indicate differences in microstructure [8]; in addition, our results show that they also allow the lithium content to be monitored, and the superstructure, formed in the intermediate phase, to be studied, as shown above.

**Figure 7.**
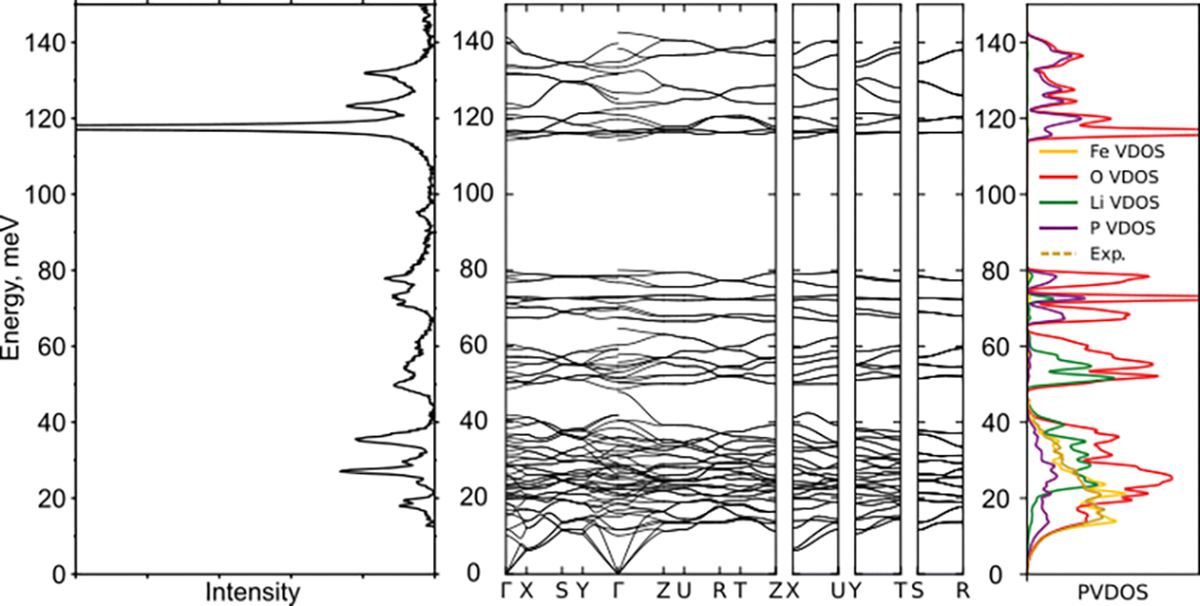
(**Left**): Raman spectrum of LFP powder; (**center**): calculated phonon band structure; (**right**): calculated element-projected PVDOS and NRVS-derived experimental Fe PVDOS.

While the Fe vibrations are not directly affected by the presence of lithium, they have strong overlap with the vibrations of oxygen and phosphorus, as shown in Figure 7. Upon delithiation, the vibrations of Fe shift to the higher energies (Figure 3), the same way as the Raman peaks [19,37], due to lattice contraction and the stiffening of Fe-O bonds.

Compared to the other vibrational spectroscopy techniques, NRVS gives valuable insights into the phonon structure of the material. It allows *operando* measurements to be performed with high temporal resolution. The acquisition of a single spectrum in our experiments took approx. 20 min, providing decent statistics to reconstruct the PVDOS with sufficient accuracy. It also does not require substantially large amounts of material and provides the component specificity (i.e., it focuses on the signal from the active material), in contrast, for example, to the inelastic neutron scattering. In addition, it provides higher energy resolution, than usually available for inelastic neutron scattering [38]. Another significant advantage is that it allows for measuring the realistic cell with minimal modifications to allow the *operando* measurement thanks to the high penetration depth of high-energy X-rays.

## 4. Conclusions

We have described the experiment to measure the vibrational properties of the active electrode material of Li-ion battery with NRVS technique. We have demonstrated that this technique is a powerful tool and is very well suited for *operando* setup, compared to other vibrational spectroscopy techniques.

It was previously shown that vibrational spectroscopy allows to detect phenomena associated with the microstructure of the material. By combining the prior structural knowledge with the experimental vibrational data, computational simulations allow us to precisely reconstruct the vibrational structure, which not only allows the various thermodynamical properties to be derived, but also to achieve insights into the atomic structure or microstructure of the material.

## Figures and Tables

**Table 1.** Atomic % concentration of the Fe isotopes of the nominal ^57^Fe iron sample as provided by the Certificate of Analysis № 240801– 089Sct by Neonest AB, issued on 1 September 2025.

Isotope:	Fe-54	Fe-56	Fe-57	Fe-58
Content (at. %):	0.005	0.615	**96.060**	3.360

**Table 2.** Occupancy parameters obtained by Rietveld refinement of XRD of LFP. Occupancy of Fe was fixed to 1.

Atom	Fraction
Fe	1
Li	0.93(13)
P	1.025(30)
O1	0.84(7)
O2	0.86(6)
O3	0.88(6)

## Data Availability

The data can be obtained upon personal request by contacting Artur Braun and Alexey Rulev.

## Abbreviations

The following abbreviations are used in this manuscript:

PDOS: Phonon density of states
NRVS: Nuclear resonance vibrational spectroscopy
LFP: Lithium iron phosphate
IR: Infrared
FP: Iron phosphate
XRD: X-ray diffraction
NMP: N-methyl-2-pyrrolidon
DOS: Density of states
PVDOS: Partial vibrational density of states
NAC: Non-analytical term correction
